# Steady-State Modulation of Voltage-Gated K^+^ Channels in Rat Arterial Smooth Muscle by Cyclic AMP-Dependent Protein Kinase and Protein Phosphatase 2B

**DOI:** 10.1371/journal.pone.0121285

**Published:** 2015-03-20

**Authors:** Jennifer L. Brignell, Matthew D. Perry, Carl P. Nelson, Jonathon M. Willets, R. A. John Challiss, Noel W. Davies

**Affiliations:** 1 Department of Cell Physiology and Pharmacology, University of Leicester, Leicester, United Kingdom; 2 Department of Cancer Studies and Molecular Medicine, Leicester Royal Infirmary, Leicester, United Kingdom; University of Texas Health Science Center, UNITED STATES

## Abstract

Voltage-gated potassium channels (K_v_) are important regulators of membrane potential in vascular smooth muscle cells, which is integral to controlling intracellular Ca2+ concentration and regulating vascular tone. Previous work indicates that K_v_ channels can be modulated by receptor-driven alterations of cyclic AMP-dependent protein kinase (PKA) activity. Here, we demonstrate that K_v_ channel activity is maintained by tonic activity of PKA. Whole-cell recording was used to assess the effect of manipulating PKA signalling on K_v_ and ATP-dependent K+ channels of rat mesenteric artery smooth muscle cells. Application of PKA inhibitors, KT5720 or H89, caused a significant inhibition of K_v_ currents. Tonic PKA-mediated activation of K_v_ appears maximal as application of isoprenaline (a β-adrenoceptor agonist) or dibutyryl-cAMP failed to enhance K_v_ currents. We also show that this modulation of K_v_ by PKA can be reversed by protein phosphatase 2B/calcineurin (PP2B). PKA-dependent inhibition of K_v_ by KT5720 can be abrogated by pre-treatment with the PP2B inhibitor cyclosporin A, or inclusion of a PP2B auto-inhibitory peptide in the pipette solution. Finally, we demonstrate that tonic PKA-mediated modulation of K_v_ requires intact caveolae. Pre-treatment of the cells with methyl-β-cyclodextrin to deplete cellular cholesterol, or adding caveolin-scaffolding domain peptide to the pipette solution to disrupt caveolae-dependent signalling each attenuated PKA-mediated modulation of the K_v_ current. These findings highlight a novel, caveolae-dependent, tonic modulatory role of PKA on K_v_ channels providing new insight into mechanisms and the potential for pharmacological manipulation of vascular tone.

## Introduction

K^+^ channels play an important role in regulating the membrane potential of vascular smooth muscle cells. Activation of K^+^ channels results in hyperpolarization, a decrease in [Ca^2+^]_i_ and vasodilation, while their inhibition leads to depolarization, an increase in [Ca^2+^]_i_ and vasoconstriction [1]. Several types of K^+^ channels are expressed in arterial smooth muscle, including ATP-dependent K^+^ (K_ATP_) channels, inward-rectifier K^+^ channels, large-conductance, Ca^2+^-activated K^+^ (BK_Ca_) channels and voltage-gated K^+^ (K_v_) channels, and all are involved in regulating the membrane potential [1–4]. K^+^ channel modulation via intracellular signalling pathways is well established [1,5]. We and others have shown that the vasoconstrictors angiotensin II (Ang-II) and endothelin-1 (ET-1) inhibit both K_ATP_ and K_v_ currents of rat mesenteric artery smooth muscle (MASMC), through activation of PKC [6,7] [8–10]. In addition to the PKC pathway, inhibition of cyclic AMP-dependent protein kinase (PKA) has also been shown to be a component of the attenuation of K_ATP_ and K_v_ current of mesenteric smooth muscle by Ang-II, suggesting a certain level of tonic activation of K^+^ channels by PKA [7,8]. Moreover, K_v_ channel activity recorded in inside-out patches of mesenteric artery smooth muscle cells is increased following application of the catalytic subunit of PKA [8].

Vasodilator-mediated activation of GPCRs, such as β-adrenoceptors, can lead to Gα_s_-mediated adenylyl cyclase (AC) activation, cAMP production and activation of PKA, leading to hyperpolarization and vasodilation. PKA-dependent enhancements of BK_Ca_ and K_ATP_ currents in pig coronary arteries by calcitonin gene-related peptide [11,12], of K_ATP_ current in mesenteric arterial smooth muscle cells by vasoactive intestinal polypeptide [13], and in rabbit portal vein and rat aortic smooth muscle by the β-adrenoceptor agonist isoprenaline [14,15] have been reported. Interestingly, however, application of agents that directly (dibutyryl-cAMP) or indirectly (forskolin) activate PKA have been shown not to enhance K_v_ currents in isolated cerebral arterial smooth muscle cells [16].

Targeting of PKA to ion channels (or associated regulatory proteins) has, in many cases, been shown to involve PKA-anchoring proteins (AKAPs) and caveolae [17,18]. AKAPs operate via a specialized anchoring domain that localizes the PKA-AKAP complex to specific intracellular locations to facilitate PKA-mediated phosphorylation [19,20]. In contrast, caveolae are invaginations that form in cholesterol- and sphingolipid-rich membrane microdomains that can be distinguished from lipid rafts by the presence of the cholesterol binding protein caveolin [18,21,22]. Signalling complexes are co-localized within these microdomains, presumably facilitating the correct targeting of signalling events [18]. Caveolin-1 and PKA have been shown to co-localize in cultured AV12 cells [23] and disruption of caveolae by cholesterol depletion uncouples AC-dependent regulation of K_ATP_ channels in vascular smooth muscle [24]. Furthermore, K_ATP_ channels expressed in HEK293 cells are inhibited, either by caveolin-1 co-expression, or by inclusion of caveolin-1 scaffolding domain peptide (CSDP) in the patch pipette [25].

Vasodilator-driven modulation of K^+^ channels via PKA signalling complexes and the role of tonic activation of PKA is poorly understood. Important yet to be defined aspects of tonic PKA modulation are, *(i)* how the catalytic PKA subunits are targeted to voltage-gated K^+^ channels, and *(ii)* what is responsible for reversing the PKA effects. In this study we have focused on investigating the extent of tonic PKA signalling and the potential targeting mechanisms for the PKA-induced activation of the voltage-gated K^+^ current in these cells. We have determined that PP2B is responsible for reversing this action of PKA and have examined the roles of AKAPs and caveolae in targeting PKA signalling to these channels.

## Materials and Methods

### Animals

All experiments were carried out on adult male Wistar rats (200–300 g) killed by cervical dislocation. The care of animals was in accordance with the UK Animals (scientific procedures) Act 1986. Investigations carried out in this study conformed to the Guide for the Care and use of laboratory animals published by the US National Institutes of Health (NIH publications No. 85–23 revised 1996). The procedures used in this study were approved by the University of Leicester Animal Care and Use Committee.

### Cell Isolation

Rat mesenteric arteries were dissected and placed in cold zero-Ca^2+^ solution containing (in mM): 5.4 KCl, 137 NaCl, 0.44 Na_2_HPO_4_, 0.42 NaH_2_PO_4_, 4 glucose, 6 mannitol, 10 HEPES and 1 MgCl_2_, adjusted to pH 7.4 with NaOH. A two-stage enzymatic digestion was then carried out at 35°C as described previously [8]. Briefly, the tissue was placed in a low-Ca^2+^ solution (the solution above with 0.1 mM CaCl_2_ added) containing 0.9 mg mL^-1^ albumin, 2.0 mg mL^-1^ papain and 2.0 mg mL^-1^ dithioerythritol for 31 min. Following this, mesenteric arteries were digested for a further 12.5 min in low-Ca^2+^ solution containing 1.1 mg mL^-1^ collagenase type F and 1.3 mg mL^-1^ hyaluronidase. The digested tissue was triturated gently to yield isolated smooth muscle cells. After isolation, cells were stored on ice in low-Ca^2+^ solution containing 1 mM sodium pyruvate for use on the same day.

### Electrophysiology

K^+^ currents were recorded from isolated smooth muscle cells using the conventional whole-cell patch clamp technique. This enabled intracellular access of membrane impermeant inhibitory peptides by their inclusion in the patch pipette. Patch pipettes (resistance 4–6 MΩ when filled) were made from filamented borosilicate glass (outer diameter 1.5 mm, inner diameter 0.86 mm; Harvard Apparatus) using a Narishige PC-10 pipette puller. Currents were recorded using an Axopatch 200A amplifier (Molecular Devices, Sunnyvale, CA, USA), filtered at 2 kHz and sampled at 10 kHz using a Digidata 1322A interface (Molecular Devices, Sunnyvale, CA, USA). K_v_ currents were activated by 400 ms voltage pulses to potentials ranging from -40 to +60 mV from a holding potential of -65 mV. A P/6 protocol was used to remove leak and capacitive currents. The intracellular solution contained (in mM): 110 KCl, 30 KOH, 10 Hepes, 10 EGTA, 1 MgCl_2_, 3.9 CaCl_2_, 1 Na_2_ATP, 0.1 ADP and 0.5 GTP adjusted to pH 7.2 with NaOH. The free [Ca^2+^], calculated using Maxchelator (http://www.stanford.edu/%7Ecpatton/maxc.html), was 100 nM. The concentrations of ATP, ADP and GTP used in the pipette were designed to give optimal activity of K_ATP_ channels whilst maintaining adequate signalling components. The external solution contained (in mM): 6 KCl, 134 NaCl, 4 glucose, 6 mannitol, 10 Hepes, 1 MgCl_2_ and 0.1 CaCl_2_, adjusted to pH 7.4 with NaOH. To minimize contamination from BK_Ca_ channel activity currents were recorded in the presence of 100 nM penitrem A, which virtually abolishes BK_Ca_ current at this concentration [26,27]. All experiments were carried out only after K_v_ currents became stable after establishing whole-cell recording [8]. Unless stated otherwise, all patch-clamp experiments were done at 30–32°C, maintained using a Dagan HW-30 temperature controller.

Recordings were analysed using pCLAMP (Molecular Devices, Sunnyvale, CA, USA) and Excel (Microsoft) software. Current-voltage curves were obtained by averaging the current between 320 and 370 ms of the test pulse and plotted either as current density (normalized to the cell capacitance) or normalized to the current value obtained at +40 or +60 mV under the appropriate control conditions.

### Materials

Caveolin scaffolding domain peptide and its scrambled version were obtained from Merck Biosciences (Nottingham, UK), calcineurin auto-inhibitory peptide was from Enzo Life Science (Exeter, UK), KT 5720 and 2’,5’-dideoxyadenosine were from Tocris (Bristol, UK) and PKA inhibitory peptide (PKI-RR, CTTYADFIASGRTGRRNAIHD) and its inactive analogue PKAi-AA were from Pepceuticals (Leicester, UK). All other chemicals and reagents were purchased from Sigma-Aldrich (Poole, UK).

### Data Analysis and Statistics

Data are presented throughout as means ± s.e.m. Statistical significance was assessed using Student’s paired *t*-test, one-way ANOVA or two-way ANOVA with Bonferroni’s *post hoc* test as appropriate. A value of *P*<0.05 was considered significant. All statistical analysis was carried out using GraphPad Prism (San Diego, CA, USA).

## Results

**Fig 1:**
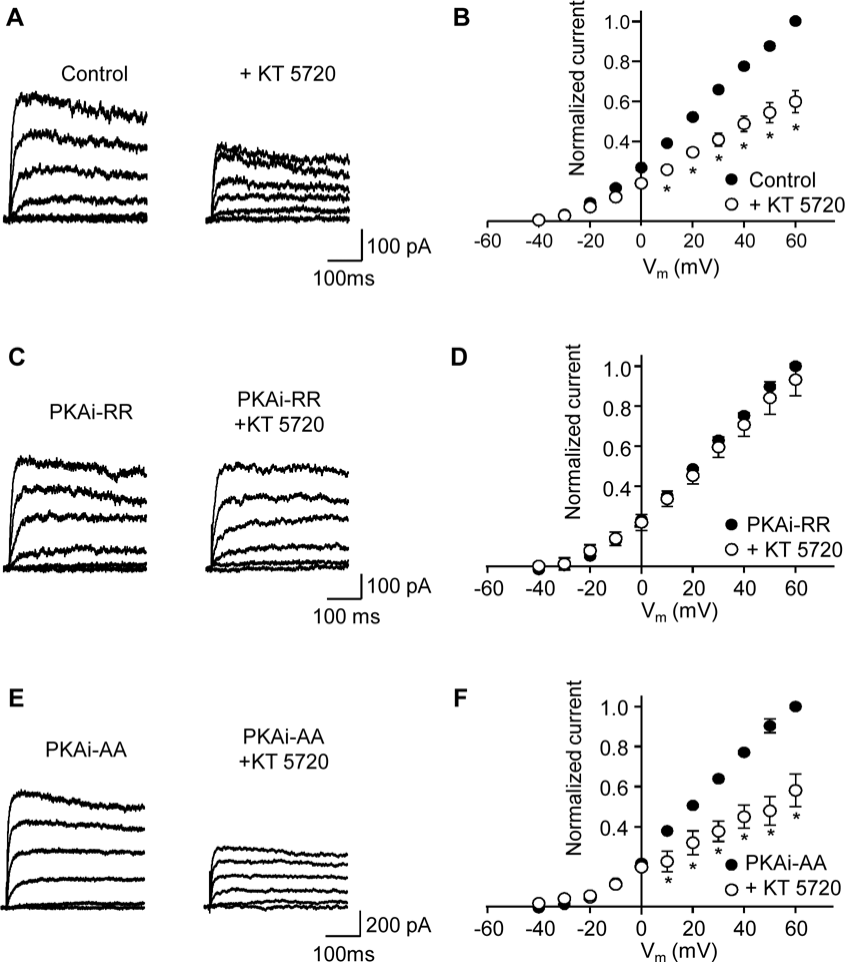
Inhibition of PKA attenuates K_v_ current. K_v_ currents were activated in response to 400 ms depolarizing voltage steps from a holding potential of -65 mV. (**A**) Representative K_v_ current traces before (control) and after application of 1 μM KT 5720 as indicated. Representative K_v_ currents shown in this and subsequent figures are in 20 mV increments beginning from -40 mV. (**B**) Mean (± s.e.m.) I-V plots (normalized to control current at +60 mV) before and after application of 1 μM KT 5720 (n = 8 cells). (**C**) Representative K_v_ current traces before and after application of 1 μM KT 5720 obtained in the presence of the PKA inhibitor peptide, PKAi-RR (5 μM) in the patch pipette. (**D**) Mean (± s.e.m.) I-V plots (normalized to the control currents, *i*.*e*. in the presence of PKAi-RR, at +60 mV) before and after application of 1 μM KT 5720 as indicated (n = 5). (**E**) Representative K_v_ current traces before and after application of 1 μM KT 5720 in the presence of PKAi-AA (5 μM), an inactive isoform of PKAi-RR, in the patch pipette. (**F**) Mean (± s.e.m.) I-V plots (normalized to the respective control currents, *i*.*e*. in the presence of PKAi-AA at +60 mV) before and after application of 1 μM KT 5720 as indicated (n = 7). *P<0.05; two-way ANOVA, Bonferroni’s *post hoc* test.

Whole-cell patch clamp recordings from rat MASMC revealed K_v_ currents activated in response to depolarizing voltage steps to potentials more positive than about -30mV (e.g. Fig. 1). These currents activated relatively slowly and displayed little inactivation during the 400 ms pulses. We found that K_v_ current density (normalized to cell capacitance) was variable in MASMC; and for this reason we have assessed the effects of pharmacological manipulation of K_v_ currents as a fraction of the currents recorded under the appropriate control conditions.

### Steady-state activation of K_v_ current by PKA

We have shown previously that a component of the Ang-II-induced inhibition of K_v_ current in rat MASMC occurs through a reduction in PKA activity [8]. To establish the level of tonic PKA activation, which would dictate the relative importance of this pathway for the regulation of K_v_ channel activity in these cells, we measured K_v_ current amplitudes before and after bath application of the PKA inhibitor KT 5720. As shown in Fig. 1A, the amplitude of the K_v_ current, measured over a range of voltages, was reduced following 10–15 min exposure to 1 μM KT 5720, indicating that under our control conditions there was considerable tonic PKA activation of K_v_ channels. Current values were normalized to the control current obtained at +60 mV for each cell and mean normalized I-V relationships are shown in Fig. 1B (n = 8). At +60 mV K_v_ current decreased to 65 ± 5% of control values following the application of KT 5720 (*P*<0.05, n = 8). A similar inhibition was found with another membrane permeable PKA inhibitor H-89 (1 μM), which decreased the current at +60 mV to 63 ± 8% of control values (*P*<0.05; n = 5). To test whether KT 5720 was blocking the current through a mechanism independent of PKA, a PKA inhibitory peptide (PKAi-RR; 5 μM) was included in the patch pipette and inhibition of the K_v_ current by KT 5720 was assessed. Mean K_v_ current densities at +60 mV, measured approximately 3 minutes following the establishment of whole-cell configurations, were 30.9 ± 3.8 (n = 13) and 21.8 ± 2.2 (n = 10) pA pF^-1^ in control and in PKAi-RR containing pipette solutions respectively (this difference was not quite significant (P = 0.06), though as stated above current densities were variable in these cells). Control K_v_ currents were recorded 3 minutes after establishing whole-cell configurations; KT 5720 was then applied and its effect on the Kv current was assessed after 10–15 minutes. Application of KT 5720 to cells recorded with PKAi-RR in the pipette no longer decreased the K_v_ current, which remained at 93 ± 8% of control current measured at +60 mV (n = 5). In contrast, KT 5720 remained effective when the pipette contained PKAi-AA, an inactive homologue of PKAi-RR; the K_v_ current was decreased to 65 ± 10% (n = 7) of the control current at +60 mV. These results are consistent with a mechanism whereby KT 5720 is reducing the K_v_ current by inhibiting PKA.

**Fig 2:**
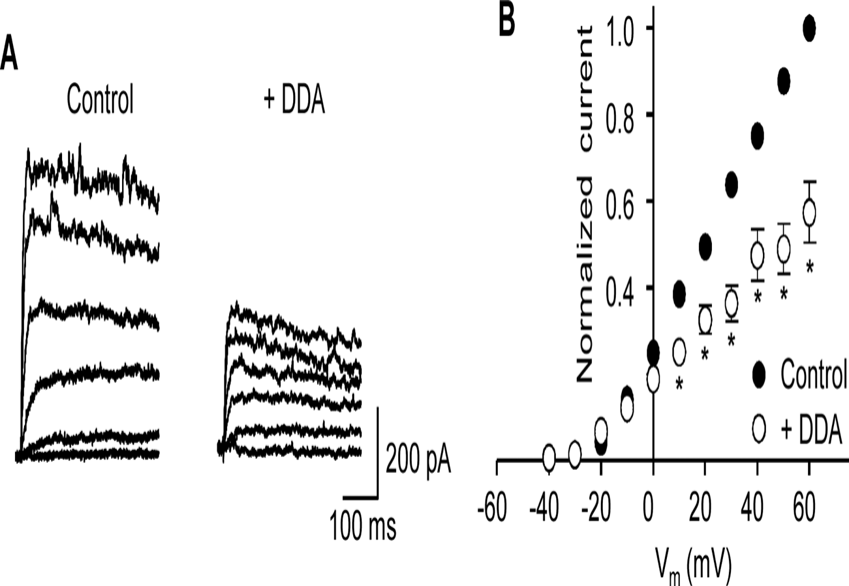
The adenlylate cyclase inhibitor, dideoxyadenosine attenuates K_v_ current. (**A**) Representative K_v_ current traces before (control) and after application of 50 μM 2’,5’-dideoxyadenosine (DDA). (**B**) Mean (± s.e.m.) I-V plots (normalized to control current at +60 mV) before (control) and after application of DDA. *P<0.05; two-way ANOVA, Bonferroni’s *post hoc* test.

Since tonic activation of PKA is likely to rely on the continued production of cAMP by AC, inhibition of AC would be anticipated to reduce PKA-dependent K_v_ channel activity. Inhibition of AC with 2’,5’-dideoxyadenosine (DDA) decreased the amplitude of the K_v_ currents recorded from MASMC (Fig. 2). K_v_ currents were measured before and 10–15 min after the application of DDA (50 μM) and normalized I-V curves were obtained as described above (Fig. 2B). Mean K_v_ current amplitude at +60 mV in the presence of DDA was 57.3 ± 2.9% of control (*P*<0.05, n = 6), suggesting that under control conditions there is significant background activity of AC.

### Activating AC failed to enhance K_v_ current amplitude

**Fig 3:**
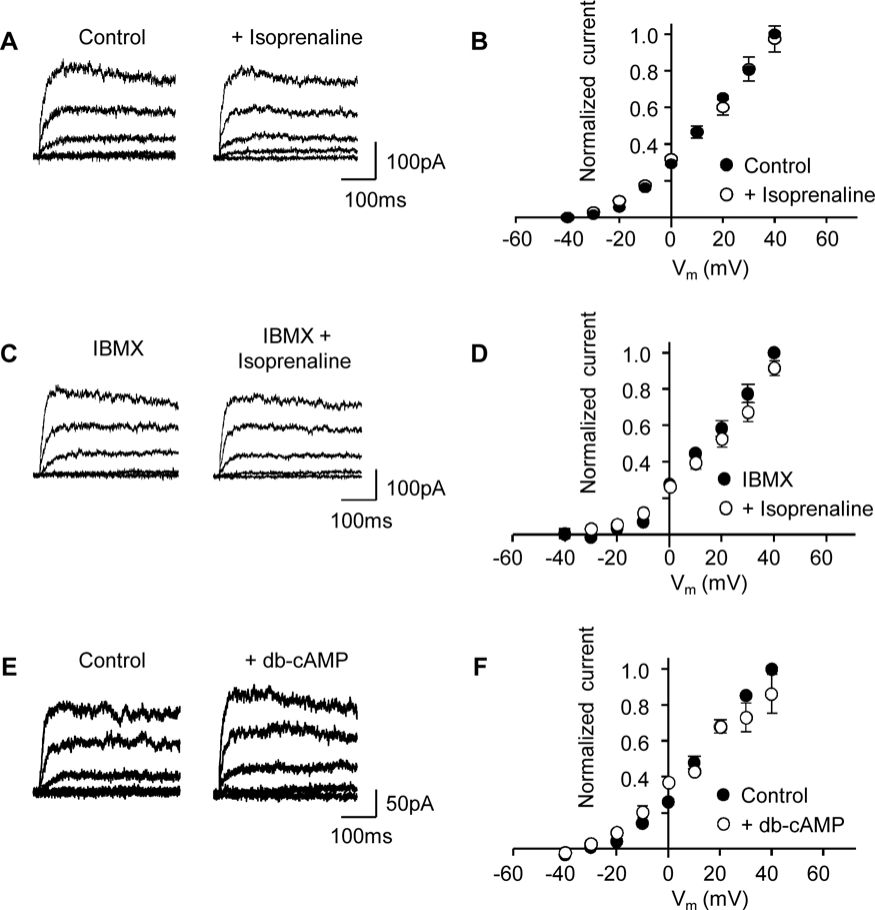
Activation of PKA does not increase K_v_ current. (**A**) Representative K_v_ current traces obtained before and after application of isoprenaline (1 μM) as indicated. (**B**) Mean (± s.e.m.) I-V plots (normalized to control current at +40 mV) before and after application of isoprenaline (1 μM; n = 8). (**C**) Representative K_v_ current traces as in panel A, but in the presence of IBMX (300 μM) before and after application of isoprenaline (1 μM). (**D**) Mean I-V plots (normalized to the control current in IBMX at +40 mV) before and after application of isoprenaline in the presence of IBMX (n = 5). (**E**) Representative K_v_ current traces before (control) and after application of dibutyryl-cAMP (db-cAMP; 100 μM). (**F**) Mean (± s.e.m.) I-V plots (normalized to the control current at +40 mV) before and after application db-cAMP (100 μM; n = 4).

**Fig 4:**
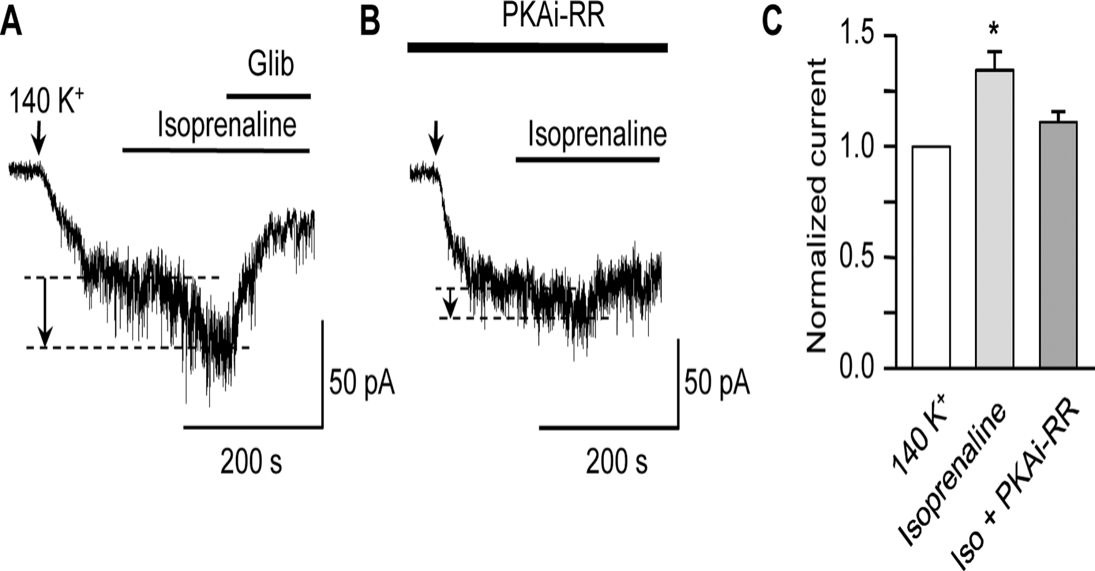
Isoprenaline activates K_ATP_ current in a PKA-dependent manner. Representative K_ATP_ current traces obtained at -60 mV in symmetrical 140 mM K^+^ following the application of isoprenaline (100 nM) in the absence (**A**) or presence (**B**) of the active PKA inhibitor peptide (PKAi-RR, 5 μM) in the patch-pipette. Arrows in this and subsequent figures indicate the point at which extracellular [K^+^] was increased from 6 to 140 mM. The current increase in response to isoprenaline is indicated by the dashed lines and arrows. (**C**) Mean K_ATP_ current, (normalized to that in 140 mM K^+^) following the application of 100 nM isoprenaline in the absence (n = 6) or presence (n = 8) of PKAi-RR in the patch-pipette (*P<0.05; one-way ANOVA, Bonferroni’s *post hoc* test).

Increasing PKA activity by vasodilators has been shown to enhance several vascular K^+^ currents [28–30], including K_v_ channels in rabbit portal vein and rat coronary artery [14,31,32]. To assess whether the K_v_ current of rat MASMC could be enhanced via GPCR-linked vasodilators we examined the effect of isoprenaline, a β-adrenoceptor agonist, on K_v_ current amplitude. Application of isoprenaline (1 μM), a concentration known to cause a >20-fold increase in cAMP in vascular smooth muscle cells [15] had little or no effect on the K_v_ currents recorded from MASMC (Fig. 3; n = 8). Little effect was observed even when isoprenaline was added to cells pre-treated for 10 min with the phosphodiesterase inhibitor IBMX (300 μM; n = 5). The lack of effect of isoprenaline on the K_v_ current was mirrored by an inability of 100 μM dibutyryl-cAMP, a membrane-permeable analogue of cAMP, to increase the K_v_ current (Fig. 3; n = 4). To check whether cAMP/PKA signalling was intact in our cells we examined whether isoprenaline, which we have shown to activate AC and K_ATP_ current in these cells [15], was still effective under our current recording conditions. K_ATP_ currents were recorded in symmetrical 140 mM K^+^ at -60 mV, which minimized contamination by K_v_ currents. In contrast to its lack of effect on K_v_ currents, isoprenaline (100 nM) effectively increased the K_ATP_ current recorded in these cells (n = 6). This increase in K_ATP_ current was dependent on PKA activation as indicated by the lack of response to isoprenaline in recordings where the patch pipette contained 5 μM PKAi-RR (Fig. 4; n = 8). These results indicated that β-adrenoreceptor stimulation was viable and able to stimulate PKA signalling to K_ATP_ channels in these experiments.

### PKA-dependent modulation of K_v_ was reversed by protein phosphatase 2B

**Fig 5:**
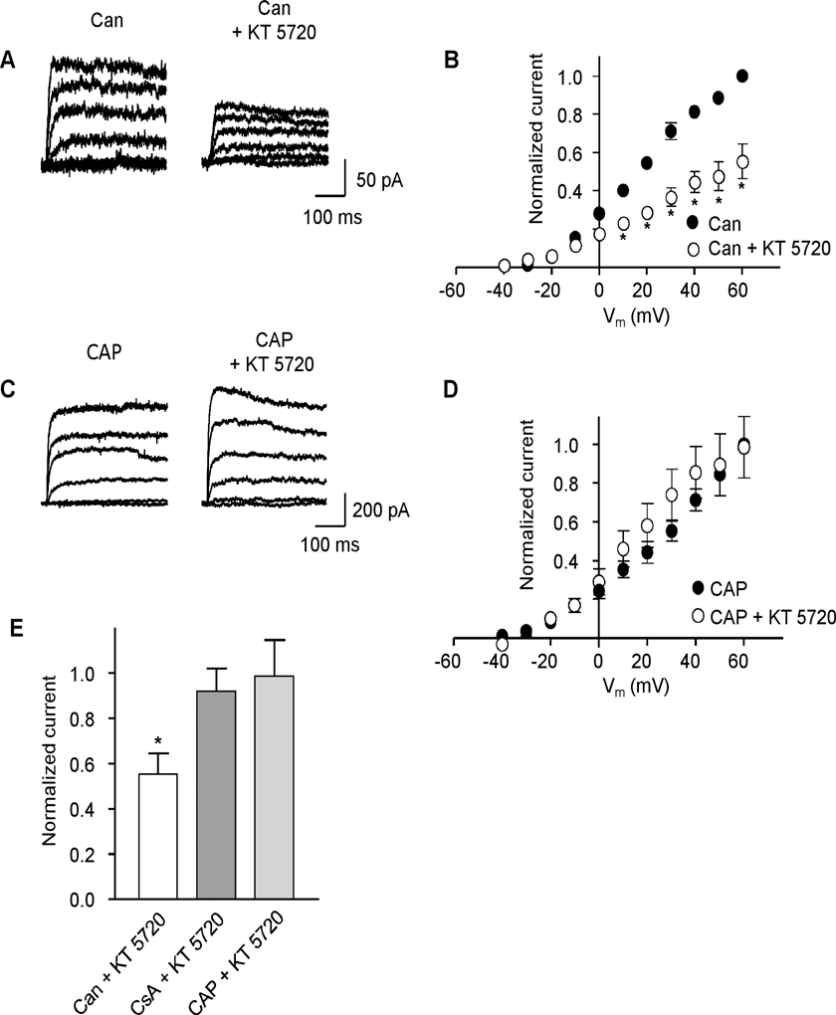
Regulation of K_v_ current in MSMCs involves protein phosphatase 2B (PP2B), but not protein phosphatase 2A (PP2A). (**A**) Representative K_v_ current traces obtained from a cell pre-treated with cantharadin (Can, 30 μM) for 10 min before and after application of KT 5720 (1 μM). (**B**) Mean (± s.e.m.) I-V plots, normalized to current in the presence of cantharadin (30 μM) at +60 mV, before and after the application of KT 5720 (1 μM, n = 4, *P<0.05; two-way ANOVA, Bonferroni’s *post hoc* test). (**C**) Representative K_v_ current traces obtained with PP2B/calcineurin auto-inhibitory peptide (CAP, 100 μM) in the patch pipette before and after application of KT 5720 (1 μM). (**D**) Mean (± s.e.m.) I-V plots, normalized to current in the presence of CAP at +60 mV, before and after application of KT 5720 (1 μM, n = 5). € Mean (± s.e.m.) current at +60 mV (normalized to respective controls) following application of 1 μM KT 5720 in cells pre-treated with cantharadin (Can, 30 μM, n = 4), cyclosporin A (CsA, 4 μM, n = 7), or with CAP (100 μM; n = 5) in the pipette as indicated (*P<0.05; one-way ANOVA, Bonferroni’s *post hoc* test).

The reduction of K_v_ current following inhibition of PKA by KT 5720 reveals that the reversal of tonic PKA activation is likely due to dephosphorylation; however, the identity of the phosphatase involved is not known. Blocking dephosphorylation should abolish the effect of inhibiting PKA, thereby removing the effect of KT 5720 on the K_v_ current. Protein phosphatases 2A (PP2A) and 2B (PP2B, calcineurin) have been found in vascular smooth muscle cells [33] and PP2B has been shown to be involved in regulation of vascular K_ATP_ channels [34]. To determine whether either of these protein phosphatases is involved in reversing the PKA-mediated modulation of MASMC K_v_ current, we examined the ability of KT 5720 to attenuate the K_v_ current in the presence of PP2A or PP2B inhibitors. Pre-treatment with the PP2A inhibitor cantharidin (30 μM for 10 min) had no effect on the reduction of K_v_ current by KT 5720 (1 μM, Fig. 5A and B). In contrast, in cells either pre-treated with the PP2B inhibitor cyclosporin A (4 μM for 10 min), or where the pipette solution contained calcineurin auto-inhibitory peptide (100 μM), KT 5720 no longer inhibited the K_v_ current (Fig. 5C and D; n = 5). These results indicate that PP2B, but not PP2A, is involved in reversing PKA-enhanced K_v_ current.

### PKA-anchoring proteins (AKAPs) do not mediate PKA activation of K_v_ currents

**Fig 6:**
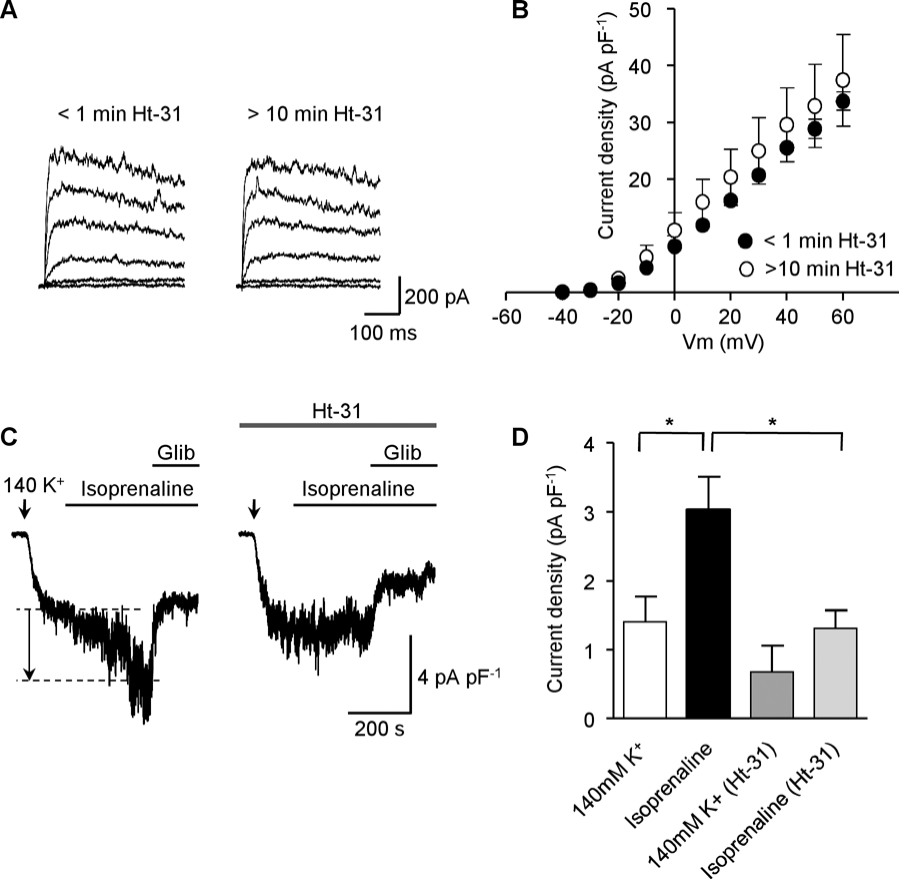
PKA activation of K_v_ currents remained following disruption of PKA-AKAP interactions. (**A**) Representative K_v_ current traces obtained immediately after establishing whole-cell recording (< 1 min Ht-31) and 10 min following establishment of the whole-cell configuration with 20 μM Ht-31 in the patch pipette. (**B**) Mean (± s.e.m.) I-V plots (current density normalized to cell capacitance) immediately after establishing whole-cell recording and 10 min after establishing whole cell configuration in the presence of Ht-31 (20 μM) in the patch pipette (n = 5). (**C**) Representative K_ATP_ current traces (normalized to cell capacitance) following the application of 100 nM isoprenaline in the absence or presence of 20 μM Ht-31 in the patch pipette; the current increase in response to isoprenaline is indicated by the dashed lines and arrow. (**D**) Mean glibeclamide-sensitive current (normalized to cell capacitance) following application of 100 nM isoprenaline in the absence or presence of 20 μM Ht-31 (n = 8 and 5 cells, respectively; *P<0.05; one-way ANOVA, Bonferroni’s *post hoc* test).

Localized PKA signalling is mediated by AKAP binding to the regulatory subunit of PKA [35], and this interaction can be abolished by Ht-31, a peptide that inhibits all PKA-AKAP interactions [36]. PKA dependent activation of K_ATP_ channels in MASMC by calcitonin gene-related peptide or by db-cAMP has been shown to be disrupted by inclusion of Ht-31 in the patch pipette [28]. We have shown already that there is considerable tonic PKA signalling maintaining the activity of K_v_ channels in these cells. If this is reliant on a PKA-AKAP interaction, inclusion of Ht-31 in the patch pipette should disrupt this, leading to a decline in K_v_ current following the establishment of the whole-cell configuration. However, we found no decline in the K_v_ current up to 10 min after the establishment of whole-cell configurations with Ht-31 (20 μM) in the pipette (Fig. 6; n = 5). In contrast, Ht-31 (20 μM) prevented the enhancement of the K_ATP_ current following application of isoprenaline (100 nM, see Fig. 6C and D). These data indicate that the interaction between PKA and AKAP, which is necessary to enable PKA signalling to K_ATP_ channels, may not be involved in PKA-mediated enhancement of K_v_ currents in MASMC.

### Caveolae are required for tonic PKA-mediated regulation of K_v_ current

**Fig 7:**
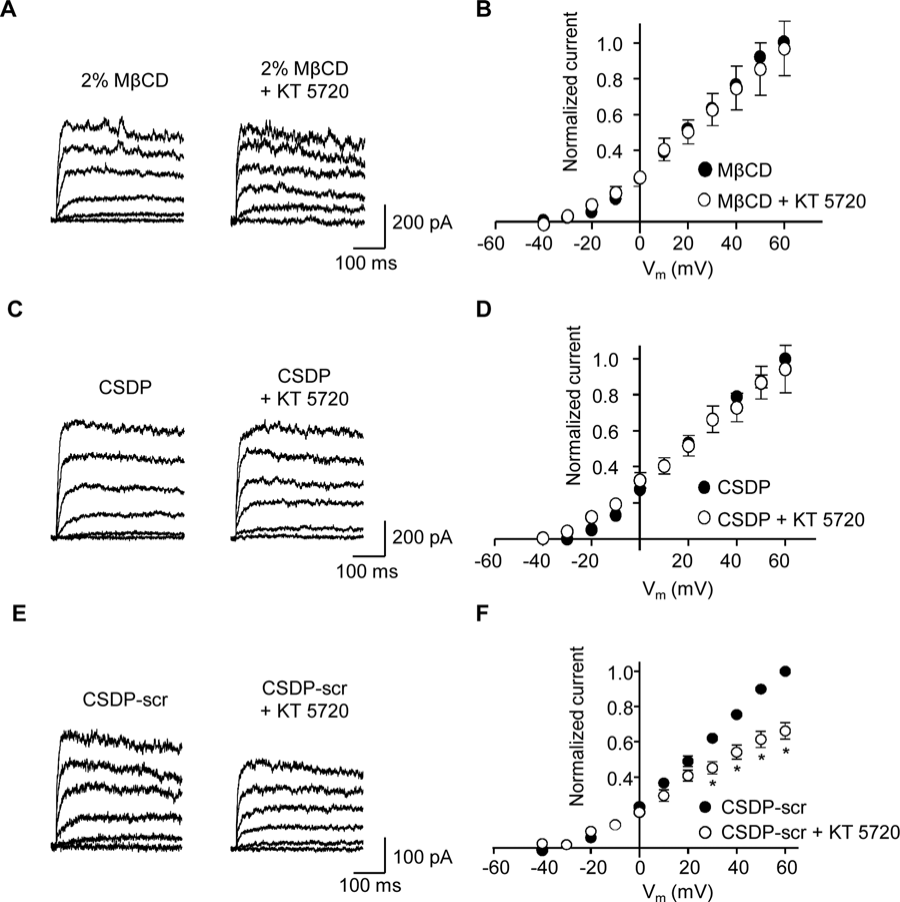
Inhibition of K_v_ current by KT 5720 is abolished by disruption of caveolae. (**A**) Representative K_v_ currents following 60 min pre-treatment with 2% methyl-β-cyclodextrin (MβCD) before and after application of 1 μM KT 5720. (**B**) Mean I-V plots (normalized to control current in the presence of MβCD at +60 mV), before and after application of 1 μM KT 5720 (n = 5). (**C**) Representative K_v_ currents in the presence of the caveolin scaffolding-domain peptide (CSDP, 100 μM) in the patch pipette, before and after application of KT 5720 (1 μM). (**D**) Mean I-V plots (normalized to the current at +60 mV in the presence of CSDP) before and after application of 1 μM KT 5720 (n = 5). (**E**) Representative K_v_ currents in the presence of a scrambled version of the caveolin scaffolding-domain peptide (CSDP-scr, 100 μM) in the patch pipette before and after application of KT 5720 (1 μM). (**F**) Mean I-V plots (normalized to the current at +60 mV in the presence of CSDP-scr) before and after application of 1 μM KT 5720 (n = 6, *P<0.05; two-way ANOVA, Bonferroni’s *post hoc* test).

Caveolae are membrane invaginations containing the cholesterol binding protein caveolin and are essential for many signalling events [18]. Caveolae have been shown to be necessary for PKA-dependent regulation of K_ATP_ channels in vascular smooth muscle [24,25], and disruption of the interaction between caveolin-1 and BK_Ca_ channels suppresses the K^+^ current in human myometrial smooth muscle cells [37]. We, therefore, investigated whether caveolae are required for tonic PKA-mediated regulation of K_v_ channels in MSMC. Caveolae are rich in cholesterol [21] and treatment with the cholesterol depleting agent methyl-β-cyclodextrin (2%; MβCD) has been shown to disrupt caveolae [38,39]. We, therefore, treated MASMC with 2% MβCD for 60 min prior to assessing the effect of KT 5720 on K_v_ currents (Fig. 7A; n = 5). In parallel experiments cells not treated with MβCD responded to KT 5720 as we show elsewhere (Fig. 1A). However, in cells pre-treated with MβCD, the K_v_ current was no longer inhibited by KT 5720 (1 μM), indicating that cholesterol depletion (and therefore disruption of caveolae) blocks PKA-dependent modulation of K_v_ current. K_v_ currents, prior to KT 5720 perfusion, in untreated versus MβCD-treated cells were not significantly different.

Since cholesterol depletion targets all lipid microdomains, to examine specifically whether caveolae were involved in PKA modulation of the K_v_ currents we used caveolin scaffolding domain peptide (CSDP) which has been shown to disrupt caveolin-1-containing complexes [25,39]. Inclusion of CSDP (100 μM) in the pipette solution blocked KT 5720-induced inhibition of K_v_ current (Fig. 7C and D; n = 5). In contrast, KT 5720-mediated inhibition of K_v_ current remained when a scrambled version of CSDP (CSDP-scr) was included in the pipette (Fig. 7E and F; n = 6). These data indicate that caveolin-1 is essential for maintaining correct PKA/PP2B signalling to K_v_ channels in MASMCs.

## Discussion

In this investigation we examined the extent of PKA-dependent modulation of K_v_ channels in MASMC. Tonic PKA-mediated activation was demonstrated by the reduction in K_v_ current following application of two structurally distinct PKA inhibitors, although the reduction was significant only at more depolarized potentials (see Fig. 1B). A reduction in current amplitude following application of the AC inhibitor DDA provides further evidence supporting tonic PKA activation of K_v_ currents in these cells. We identified the protein phosphatase that opposes PKA phosphorylation as PP2B, since blocking PP2A with cantharidin had no effect on the ability of the PKA inhibitor KT 5720 to reduce K_v_ currents whereas blocking PP2B, either with cyclosporin A, or by including calcineurin inhibitory peptide in the patch pipette, rendered the K_v_ current insensitive to KT 5720. Finally, our results indicate that caveolae are a necessary component of the PKA/PP2B-dependent modulation of K_v_ in MASMC.

Many K^+^ channels of vascular smooth muscle, including K_v_ channels, are subject to modulation by protein kinases [5]. We and others have shown that K_v_ channel activity in several vascular beds is reduced by vasoconstrictor-induced activation of PKC [6,8,10]. In addition to PKC activation, a component of the Ang-II-induced reduction of MASMC K_v_ current occurs through inhibition of PKA [8]. Although the extent of tonic PKA activation was not examined, Hayabuchi and colleagues did show direct activation of K_v_ channels following application of catalytic subunits of PKA to excised patches [8], as has been reported also for patches excised from rabbit portal vein myocytes [14]. Similarly, application of the β-adrenoceptor agonist isoprenaline to stimulate AC was shown to enhance the K_v_ current in rabbit portal vein in a PKA-dependent manner [32]. Surprisingly, neither application of isoprenaline (even following the inhibition of endogenous phosphodiesterase activity by IBMX) nor direct activation of PKA by db-cAMP was able to increase K_v_ currents of rat MASMC in our investigations. Comparable to our findings, application of forskolin or db-cAMP to rat cerebral artery cells revealed a similar inability of PKA activation to enhance K_v_ currents [16]. Although Li *et al*. [31] observed an increase in K^+^ currents in response to isoprenaline in coronary SMCs, the currents measured included BK_Ca_ activity and therefore do not definitively demonstrate an enhancement of K_v_ current. To confirm the viability of PKA signalling in our cells we examined the effect of isoprenaline on the K_ATP_ current. An increase in K_ATP_ current following receptor-linked activation of AC, has been shown previously in rat MASMCs exposed to calcitonin gene-related peptide [28] or isoprenaline [15], and in pig coronary arteries exposed to adenosine [40]. We also observed an increase in K_ATP_ current following application of isoprenaline to MASMC, which did not occur when PKAi-RR was in the pipette, demonstrating functioning PKA signalling to K_ATP_ channels in our cells. We can therefore conclude that the absence of any enhancement of K_v_ current in response to isoprenaline (or other cAMP-elevating manipulations) reflects an insensitivity of this channel to further AC/PKA activation.

Previous work has shown that neuronal K_v_2.1 channels can be dephosphorylated by calcineurin [41], and ceramide which inhibits Kv currents in pulmonary and mesenteric arteries [42], has been shown to activate protein phosphatase 2A [43]. These findings suggest that K_v_ channels could be modulated by PP2A and PP2B. Here we show that the protein phosphatase involved is PP2B, since blocking PP2A with cantharidin had no effect on the ability of KT 5720 to reduce K_v_ currents, while blocking PP2B, either with cyclosporin A, or by inclusion of calcineurin inhibitory peptide in the patch pipette, rendered the K_v_ current insensitive to KT 5720. Indeed, an opposing regulation of ion channels by PKA and calcineurin has been previously established in other systems. For example, Santana *et al*. [44] showed that modulation of calcium channels in cardiac myocytes was mediated via PKA and calcineurin. L-type Ca^2+^ channels in L6 myocytes are inhibited by PKCα induced phosphorylation; however, a slight increase in intracellular [Ca^2+^] removed this inhibition which was restored by blocking PP2B [45]. These authors proposed that a low and maintained release of Ca^2+^ from intracellular stores activated PP2B which reversed the PKC induced phosphorylation. Interestingly, K_ATP_ channel modulation in both aortic smooth muscle and a heterologous expression system occurs via PP2B [34,46]. It would, therefore, be interesting to investigate whether there is a common mechanism for dephosphorylation of steady-state activation of both K_v_ and K_ATP_ in vascular smooth muscle via PP2B. However, the balance between phosphorylation and dephosphorylation is likely to reflect more complex interactions, as a similar experimental approach used by Mason *et al*. [47] revealed that PKA-activated K_v_1.5 channels expressed in *Xenopus* oocytes were dephosphorylated by a protein tyrosine phosphatase and not PP2A or PP2B. Also both PKA and PP2B have been shown to bind to AKAP79, though at different sites [48,49]. If PP2B interacts with AKAPs in our preparation, it is possible that disrupting AKAP function with Ht-31restricts PP2B induced dephosphorylation. Thus, the regulation of K_v_ channel phosphorylation and dephosphorylation revealed in this study may reflect only an aspect of the intracellular signalling between PKA, PP2B and K_v_ channels.

Both caveolae and AKAPs are necessary to direct PKA signalling to vascular K_ATP_ channels. The AKAP inhibitor Ht-31 effectively uncouples steady-state PKA activation of K_ATP_ channels in rat MASMC demonstrating that AKAPs are necessary to maintain PKA signalling to K_ATP_ channels in these cells [24,28]. In contrast to the effects on K_ATP_ current, we found that Ht-31 peptide had little effect on the ability of KT 5720 to attenuate K_v_ current, suggesting that steady-state PKA activation of MASMC K_v_ channels is not dependent on AKAPs. A potential drawback to disrupting AKAP-PKA complexes is a general increase in cytosolic PKA and potentially a resultant abnormal PKA-driven phosphorylation of substrates [35]. A recent report has indicated that the scaffolding protein PSD95 is necessary for PKA signalling to K_v_1.2 channels in rat cerebral arteries [50] and it would be interesting to test whether this scaffolding protein too has a role in targeting PKA phosphorylation of K_v_ channels in MASMC. However, cholesterol depletion, which is known to disrupt caveolae [39], rendered the K_v_ current insensitive to KT 5720, suggesting that intact caveolae are necessary to maintain proper PKA and/or PP2B targeting to K_v_ channels in our cells. Caveolae are identified by the presence of the cholesterol binding protein caveolin, the scaffolding domain of which interacts with many caveolae-associated channel proteins [18]. We found that inclusion of CSDP [51] in the pipette disrupted the ability of KT 5720 to reduce K_v_ current amplitude, consistent with a mechanism where interaction of K_v_ channels and PKA require intact caveolae. Co-expression of caveolin-1 and PKA led to a punctate co-localization, while expression of PKA alone resulted in a diffuse expression of PKA [23]. K_v_1.5 channels have been shown to interact with caveolin-1 to form a signalling complex with 5-HT receptors in pulmonary artery smooth muscle [52] and specific trafficking of K_v_1.5 subunits to cholesterol-rich membrane domains requires caveolin [38]. These findings, along with the observations presented here, suggest that caveolar localization is crucial for the physiological regulation of K_v_ channel activity.

In conclusion, our results show that there is a tonic PKA activation of K_v_ currents in rat MASMC which is reversed by the action of PP2B. This signalling is dependent on the presence of functional caveolae and on an interaction with caveolin-1. Activation of AC with isoprenaline, or direct activation of PKA by db-cAMP, did not increase K_v_ current further. This suggests that the amount of PKA-induced phosphorylation leading to K_v_ channel activation is maximal and overcomes dephosphorylation by PP2B, which is revealed only in the presence of PKA inhibition. Alternatively, additional PKA activity induced by isoprenaline, which causes a PKA-dependent increase of K_ATP_ current, may not be targeted to enhance K_v_ current. A precedent for such targeting of signalling pathways to K_v_ channel modulation in MASMCs is provided by work with ET-1 and Ang II [10,53]. While both vasoconstrictors activate PKCα and PKCε [53] inhibition of K_v_ current by ET-1 occurs only through PKCα, while Ang II-mediated inhibition occurs only through PKCε. The evidence presented in the present study indicates that AC/PKA-mediated signalling to K_v_ channels is also tightly regulated. The subunit composition of K_v_ channels within vascular smooth muscle is complex, and pharmacological and biophysical evidence suggests that the K_v_ current is a composite passing through channels comprising different homo- or heteromultimers [54]. Varying expressions of several K_v_α subunits (including K_v_1.2, 1.3, 1.5, 2.1 and K_v_7.1–7.5) and K_v_ß subunits (K_v_ß1.1, 1.2 and 1.3) have been detected in smooth muscle from different vascular beds [55, 56–58]. We focused our current study on assessing the level of tonic PKA activation of the overall K_v_ current and on identifying the protein phosphatase involved in reversing this effect. Future experiments shall focus on identifying the relative role of PKA phosphorylation of specific subunits should enhance our understanding of K_v_ channel modulation by PKA. Given the central role played by K_v_ in regulating myogenic tone steady-state modulation of K_v_ by PKA is likely to play a pivotal role in determining blood flow and pressure in the resistance vasculature [50,55].
